# Prostaglandin F2 Alpha Triggers the Disruption of Cell Adhesion with Cytokeratin and Vimentin in Bovine Luteal Theca Cells

**DOI:** 10.3390/ani11041073

**Published:** 2021-04-09

**Authors:** Sang-Hee Lee, Seunghyung Lee

**Affiliations:** 1 School of Information and Communications Technology, University of Tasmania, Hobart, TAS 7001, Australia; sang1799@kangwon.ac.kr; 2 Institute of Animal Resources, Kangwon National University, Chuncheon 24341, Korea; 3 College of Animal Life Sciences, Kangwon National University, Chuncheon 24341, Korea

**Keywords:** ovary, corpus luteum, luteolysis, cell adhesion, prostaglandin F2 alpha

## Abstract

**Simple Summary:**

Luteolysis is an important event in the control of the corpus luteum function in bovines. However, some aspects of the luteolytic mechanism remain unclear. We evaluated changes in cell adhesion in luteal cells during regression of corpus luteum. Bovine luteal theca cells (LTCs) were treated in vitro with Prostaglandin F2 alpha (PGF2α). Cytokeratin, vimentin and desmoplakin proteins in LTCs were disrupted by PGF2α, affecting cell adhesion. These results suggest that PGF2α plays an important function in cell adhesion during the regression of corpus luteum.

**Abstract:**

Intermediate filaments (IFs) maintain cell–cell adhesions and are involved in diverse cellular processes such as cytokinesis, cell migration and the maintenance of cell structure. In this study, we investigated the influence of prostaglandin F2 alpha (PGF2α) on cytokeratin and vimentin IFs, Rho-associated protein kinase (ROCK), and cell-cell adhesion in bovine luteal theca cells (LTCs). The luteal cells were isolated from bovine corpus luteum (CL), and the LTCs were treated with 0, 0.01, 0.1 and 1.0 mM PGF2α. Cytokeratin, vimentin and desmoplakin proteins were disrupted and the ROCK protein was significantly increased in PGF2α-treated LTCs. In addition, cell–cell adhesion was significantly (*p* < 0.05) decreased in the PGF2α-induced LTCs compared to control group (0 mM PGF2α). In conclusion, PGF2α affected the adhesion of cell to cell via disruption of desmoplakin, cytokeratin and vimentin, additionally increasing ROCK in bovine LTCs. These results may provide a better understanding of the mechanism of bovine CL regression.

## 1. Introduction

The corpus luteum (CL) is a temporary endocrine organ that synthesizes progesterone (P4) to establish and maintain pregnancy, and it repeatedly undergoes formation (luteogenesis) and regression (luteolysis) during the estrous cycle [1]. Formation of the CL is initiated by the ovulatory surge of luteinizing hormone (LH) and proliferation and adhesion of ovary cells are actively accompanied by angiogenesis [1]. The CL is composed of a heterogeneous populations of cells that are largely classified into two categories according to function: luteal steroidogenic cells (LSCs) and non-LSCs such as luteal endothelial cells (LECs), pericytes, fibroblasts and lymphocytes [2]. Furthermore, the interaction of these cells is not only responsible for the steroidogenic functions, but also for maintaining tissue architecture by tight junctions, gap junctions, adherens junctions, and desmosomes [3,4,5,6]. Therefore, it is necessary to study cell–cell adhesion to understand maintenance and degradation in CL structure.

In general, LSCs are classified as large and small according to their size, with large LSCs originating from granulosa cells (GCs), whereas thecal cells (TCs) differentiate into small LSCs [7]. In addition, large and small LSCs are also referred to as luteal GCs (LGCs) and luteal TCs (LTCs), and they have different characteristics, such as the amount of P4 production and cellular surface molecules [8,9,10,11,12]. The roles of GCs and LGCs in follicular and luteal function have been the targets of intensive research, with much more interest spent on these cells compared to investigations of the roles of TCs and LTCs, probably due to greater availability of GCs for in vitro experiments, particularly on the major site of gonadotrophin action and estrogen and P4 production in follicular and luteal function [13].

The cytoskeleton is present in the cytoplasm of all eukaryotic cells; its primary function is to give the cell its shape and physical resistance to deformation, and through interconnection with the extracellular matrix and other cells the cytoskeleton stabilizes entire tissues [14]. Eukaryotic cells contain three main kinds of cytoskeletal filaments, microfilaments, microtubules, and intermediate filaments (IFs), and they are connected with cell adhesion molecules (CAMs) on the plasma membrane, which are essential for cell–cell adhesion [15]. Cell–cell adhesions are divided into tight junctions, adherens junctions, gap junctions and desmosomes; of these, the adherens junction and desmosome connections are maintained through anchoring plaques connecting actin filaments, IFs and CAMs [14]. IFs are 0.5–7.0 μm in thickness and maintain desmosomes through the combination of desmoplakin, desmoglein, and desmocollin. Vimentin, cytokeratins, and desmin are IFs that are normally and selectively expressed in ovary epithelium, stroma cells, endothelium, oocytes, GCs and TCs, and cytokeratin 18 and 19 are selectively expressed depending on the LEC morphology in cows [16,17]. Moreover, studies on cytokeratin expression have reported that the ratio of cytokeratin 8/18 protein-positive LCs was higher on days 5–6 than on day 18 after ovulation, and cytokeratin 8/18-negative LCs had increased apoptosis in cows [18]. However, the study of vimentin and cytokeratin via the desmosome has not been clearly demonstrated in bovine LCs.

The Ras homologous (Rho) protein is a member of the Ras superfamily of small GTPases and is primarily associated with cytoskeleton regulation [19]. The Rho proteins cycle between an inactive guanosine diphosphate (GDP)-bound conformation and an active guanosine triphosphate (GTP)-bound conformation that act as molecular switches to regulate a variety of cellular processes, including cell migration, adhesion, polarity and cytokinesis [20,21]. A large number of upstream regulators, categorized as GTPase activating proteins (GAPs) and guanine nucleotide exchange factors (GEFs), regulate Rho GTPases, and of these regulators, RhoGEF is activated by G protein-coupled receptors (GPCRs) on the plasma membrane [20]. According to the cellular process, the regulators increase the RhoGTP conformation, and finally Rho-associated protein kinase (ROCK) is activated [20,21]. ROCK regulates depolymerization, progression of cytokinesis and neurite retraction of glial fibrillary acidic protein (GFAP), vimentin, and neurofilament (NF-L) in mammalian cells [22,23,24]. Thus, activation of GPCRs controls phosphorylation of IF proteins via Rho/ROCK [25].

In the absence of maternal recognition between the embryo and uterus, the CL is regressed by prostaglandin F2 alpha (PGF2α), and then the ovary initiates a new reproductive cycle [1]. At the cellular level, GPCRs (PGF2α receptors) on the membranes of LSCs and LECs activate G-proteins by PGF2α, which leads to apoptosis of LCs [26,27,28], and in cows PGF2α is a key factor for apoptosis or programmed cell death in the structural regression of luteal tissues and cells [29,30]. Additionally, studies on interactions between extrinsic apoptotic (Fas/FasL, tumor necrosis factor-α, and interferon-γ) and intrinsic apoptotic (Bax and Bcl-2) signaling cascades in structural luteolysis have been performed in bovine luteal cells [30,31,32]. Comprehensively, previous studies on extrinsic and intrinsic signaling cascades in PGF2α-induced or spontaneous luteolysis in luteal cells and tissues have ultimately resulted in caspase family activation that leads to structural regression in the CL [33]. Apoptosis is activated by caspase proteins that affect not only the cleavage of various actin filament molecules but also disrupt cytokeratin and vimentin; eventually, cell-cell adhesions are disconnected due to disruption of adhesion junctions and desmosomes that are involved in actin filaments and IFs [34,35,36]. Similarly, studies on the influence of PGF2α on caspase activation in bovine LSCs and the relation between apoptosis and IF disruption in various mammalian cells have been widely reported; however, the role of PGF2α on IFs and desmosomes has not yet been clearly demonstrated in bovine LSCs. Moreover, even though cytoskeleton disruption is closely associated with Rho/ROCK signaling, most studies regarding cytoskeleton disruption in bovine LSCs have focused on extrinsic and intrinsic apoptotic signaling cascades. From a histological perspective, the CL is composed of a heterogeneous cell mixture, and the LTCs are closely associated with adhesion of heterogeneous cells and connections between heterogeneous cells in the CL [37]. Therefore, this study investigated the influence of PGF2α on Rho/ROCK activities, IFs, desmosomes and cell-cell adhesion in bovine LTCs.

## 2. Materials and Methods

### 2.1. Experimental Design

To investigate influences of PGF2α on Rho/ROCK activities, IFs, desmosomes and cell–cell adhesion in bovine CLs, firstly, we observed the luteal cells in the secretion- and regression-phases of CLs using histological assay. Secondly, the LTCs were isolated from secretion-phase CLs and thirdly we investigated mRNA regarding surface marker, steroidogenic, and cytoskeleton in the LTCs. Fourthly, we observed influence of the PGF2α on IFs (cytoskeleton and vimentin) using Immunofluorescence methods and, fifthly, we investigated change of the Rho and ROCK proteins in the PGF2α-induced LTCs to understand the Rho/ROCK relationship and factors regarding cell–cell adhesion. Next, we detected protein regarding desmosome (desmoplakin) in the PGF2α-induced LTCs, and finally investigated the capacity of the cell–cell adhesion of the PGF2α-induced LTCs.

### 2.2. Animal Ethics and Tissue Collection

All procedures involving animals were approved by the Kangwon National University Institutional Animal Care and Use Committee (KIACUC-09-0139). The CLs were collected from five slaughtered *Korean Native Cattle* heifers (28.4 ± 1.1 month) in the slaughterhouse (Jeail Industry, Hongcheon, Korea), ant transferred to the laboratory within 2 h at 4 °C. The CL samples were classified as 12–15 days (secretion-phase) and 18–20 days (regression-phase) after ovulation according to previous study [38]. Five secretion-phase CLs and five regression-phase CLs were used for the histological experiment, and another five secretion-phase CLs were only used to isolate LTCs.

### 2.3. Histological Analysis

The secretion-CLs (*n* = 5) and regression-phase CLs (*n* = 5) were fixed in 10% formalin (Sigma, St. Louis, MO, USA) for 24 h at room temperature (RT) after samples were dehydrated by ethanol and xylene, embedded in paraffin. Samples were sliced in 4 μm sections using a microtome, then were deparaffinized in xylene and rehydrated in ethanol solutions (100%, 90%, 80%, and 70%) for 5 min per each step. The samples were stained in hematoxylin solution (Sigma) for 5 min, washed with distilled water for 10 min, and stained in EosinY solution (Sigma) for 1 min. Then samples were washed with distilled water, and dehydrated in ethanol (70%, 80%, 90% and 100%) and 100% xylene for 5 min per each step. The slides were mounted with Histomount Mounting Solution (Thermo Fisher, Waltham, MA, USA), then evaluated using microscope (BX-50, Olympus, Tokyo, Japan).

### 2.4. Isolation of LTCs

The GCs were isolated to compare steroidogenic function with LTCs from large follicles of ovaries (*n* = 5) and LSCs and LECs were collected from secretion-phase CLs (*n* = 5) [39,40]. The secretion-phase CL tissues were chopped and enzymatically dissociated to isolate mixed luteal cells (LCs) in Dulbecco Modified Eagle medium (DMEM; Sigma) containing 0.65 mg/mL collagenase A (Thermo Fisher), 50 U/mL DNase I (MGmed, Seoul, Republic of Korea) and 0.1% BSA at 30 °C for 90 min, then 10% (*v*/*v*) DMEM/Nutrient Mixture F12 (D/F12; Sigma) containing 10% fetal bovine serum (FBS; Welgene, Seoul, Republic of Korea) was mixed for 5 min. The suspensions were filtered through a cell strainer (100 μm pore size, Sigma), and washed three times using the D/F12 containing 10% FBS and 1% penicillin/streptomycin solution (P/S; Sigma) at 630 g, 440 g, and 330 g for 10 min at 4 °C. The LCs pellet was seeded in a culture dish (Nunc, Roskilde, Denmark) and cultured in D/F12 containing 10% FBS and 1% P/S (culture media) at 38.5 °C, 5% CO_2_. After 24 h seeding, attached LCs were used for experiment, then to isolate LTCs the LCs were seeded at 1.0 × 10^4^ cells/mL in culture media at 38.5 °C, 5% CO_2_. After 24 h, LTCs colonies were separately collected using cloning cylinders (Thermo Fisher) according to mesenchymal cell morphology under microscope (TS-100, Nikon, Tokyo, Japan), then centrifuged at 400× *g* for 5 min; then cultivated LTCs were used for experimental PGF2α treatment.

### 2.5. Flow Cytometric Analysis

The LCs and LTCs were dissociated to single cells using 0.25% Trypsin-EDTA and the samples were centrifuged at 400× *g* for 5 min. A total of 1.0 × 10^6^ cells was resuspended in 1 mL PBS containing 4% formaldehyde (Sigma) and incubated for 10 min at 38.5 °C, then samples were incubated for 1 min at 4 °C. The suspension was centrifuged at 400× *g* for 5 min, resuspended in 1 mL 90% methanol, incubated for 30 min at 4 °C, and we removed the 90% methanol at 400× *g* for 5 min. Then 1 mL PBS containing 1% BSA (PBS/BSA) was added in samples, and incubated for 30 min at 4 °C. Samples were centrifuged at 400× *g* for 5 min, then samples were incubated in 1 mL 3% BSA/PBS containing β-actin mouse monoclonal IgG (1:250, sc-47778, SCBT) for 1 h at RT, after being washed twice (400× *g* and 5 min) using 3% BSA/PBS solution. The samples were incubated in 1 mL 3% BSA/PBS containing mouse-IgGκ binding protein-conjugated to fluorescein (BP-FITC) (1:250, sc-516140, SCBT) for 30 min at RT in the dark room, after being washed twice using 1% BSA/PBS solution. The cells were resuspended in 1 mL PBS, and analyzed via flow cytometry (FACSCalibur, BD Bioscience, San Jose, CA, USA) using argon laser tuned to 488 nm. The morphological categorization in LCs was conducted according to subpopulation area via FSC and SSC dot-plot data only in β-actin positive cells. Then comparison of relative cellular size between red blood cells, LGCs, LTCs, LECs and others was analyzed using *X*-axis values peak in FSC histogram data, and all flowcytometric data were analyzed using CELLQuest software (BD Bioscience). The detecting methods of the LCs populations were as referred to in previous studies [9,10].

### 2.6. PGF2α Treatment in LTCs

LTCs were pre-incubated for 24 h, the cells washed twice with PBS containing 0.1% BSA and the medium was replaced with fresh medium containing phenol red free D/F12 (Sigma) supplemented with 0.1% BSA, 1% P/S solution, and treated with 0, 0.01, 0.1 and 1.0 mM PGF2α (sc-203219, SCBT, Santacruz, CA, USA) for 24 h. The minimum concentration of PGF2α to induce regression in bovine LTCs was determined according to previously studies [41,42].

### 2.7. Quantitative PCR

**Figure 1:**
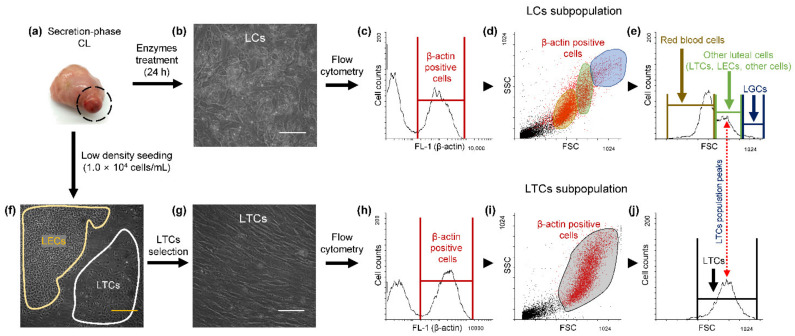
Isolation of bovine luteal theca cells (LTCs) and detection of morphology using flow cytometry. The corpus luteum (CL) tissues ((**a**), black cycle) were used at 12–15 days after ovulation and luteal cells ((**b**); LCs) and LTCs ((**f**), white area, and (**g**)) were used for experiment. The LCs and LTCs were stained by β-actin antibody conjugated with conjugated to fluorescein (FITC) dye to distinguish debris from the LCs (**c**,**h**). The subpopulation of β-actin-positive LCs ((**d**), red dots) were largely classified into three areas: red blood cells ((**d**), yellow area), other luteal cells ((**d**), green area) and luteal granulosa cells (LGCs; (**d**), blue area). The relative size of the LCs was analyzed using histogram data of flow cytometry (**e**). The subpopulation of the β-actin-positive LTCs ((**h**,**i**), red dots) was observed. The maximum peaks of *x*-axis of LTs (**e**) and LTCs (**j**) histogram data were matched (Red arrow). White scale bar: 100 μm and yellow scale bar: 400 μm.

The cultivated GCs from follicles were collected using Trizol (Takara, Shiga, Japan) and LCs (Figure 1b), LTCs (Figure 1g), and LECs (Figure 1f, yellow line area) from secretion-phase CLs were also dissociated using TRIzol (Takara) from culture dishes. The part of the secretion-phase CLs, which were used to isolate LTCs, were gathered into Trizol (Takara) and these samples were used as positive control in the experimental steroidogenic verification. All processes of the extraction mRNA using Trizol (Takara) were followed according to product manual. The mRNA concentration was measured using a NanoDrop 2000 spectrophotometer (Thermo Fisher). The mRNA was extracted using TRIzol, then total 5.0 μg mRNA was transcripted to cDNA using PrimScript 1st strand cDNA synthesis kit (Takara), and reverse transcription was performed at 45 °C for 60 min after 95 °C for 5 min. The 1.0 μL synthesized cDNA were used to conduct PCR according to the primer conditions (Table 1) using PCR premix kit (Bioneer, Seoul, Republic of Korea). The PCR products were separated with 2.0% aga-rose gel electrophoresis at 100 V for 20 min and visualized with ethidium bromide (Sigma) and UV light. The PCR product expression was analyzed with ImageJ software (NCBI, USA).

### 2.8. Western Blotting

0 and 0.01 mM PGF2α-induced LTCs were washed three times using PBS and Mammalian Protein Extraction Reagent (Thermo Fisher) and resuspended for extraction of the proteins. All processes of protein extraction were followed according to the instructions of the manufacturer. The proteins (25 μg/20 μL) were separated by sodium dodecyl sulfate–polyacrylamide gel electrophoresis (SDS-PAGE) at 30 V for 20 min after 100 V for 90 min, transferred to a polyvinylidene difluoride (PVDF) membrane at 30 V for 180 min at 4 °C, and incubated in tris-buffered saline containing 5% skim milk and 0.5% Tween-20 (TBS-T) at RT for 60 min. The PVDF membranes were incubated with TBS-T with 1% BSA containing Rho (1:1000, sc-418, SCBT), ROCK (1:1000, sc-17794, SCBT), and β-actin (1:1000) mouse monoclonal IgG antibodies at 4 °C for overnight. The membranes were washed three times with TBS-T every 5 min and incubated with goat-anti-mouse IgG-HRP (1:5000, sc-2005, SCBT) for 1 h at RT after washing, then visualized using the West Save Enhanced Chemiluminescence kit (AbFrontier, Austin, TX, USA). Protein expression was measured using the EZ-Capture II system (ATTO, Tokyo, Japan) and protein band intensity was calculated using ImageJ software.

### 2.9. Immunofluorescence

The LTCs (1.0 × 10^4^ cells/mL) were incubated in the culture media using a Chamber Slide system (Thermo Fisher) for 24 h, and the LTCs were washed twice with 0.1% BSA/PBS, after being cultured in phenol red free D/F12 (Sigma) supplemented with 0.1% BSA, 1% P/S, and PGF2α for 24 h. Then the LTCs were washed twice with 0.1% BSA/PBS and were incubated for 30 min using 0.1% BSA/PBS supplemented 3% paraformaldehyde (Sigma). After twice washing in 0.1% BSA/PBS, the LTCs were incubated for 20 min in 0.1% BSA/PBS supplemented 0.25% Triton-X (Sigma). The LTCs were incubated in 1% BSA/PBS for 30 min at RT, and after washing the cells were incubated with 0.1% BSA/PBS supplemented cytokeratin (sc-32329, SCBT), vimentin (sc-32322, SCBT) and desmoplakin (sc-390975, SCBT) mouse monoclonal IgG antibodies for 3 h at RT and all primary antibodies were diluted with 0.1% BSA/PBS until 1:100. After washing twice, the LTCs conjugated with cytokeratin and desmoplakin antibodies were incubated with goat anti-mouse IgG-FITC (1:400, Cat. 62-6511, Thermo Fisher) and conjugation with vimentin was incubated with goat anti-mouse IgG-Alexa Fluor 546 (1:400, Cat. A-11030, Thermo Fisher) for 1 h at RT in dark room. After twice washing, 0.1% BSA/PBS supplemented 300 nM DAPI (Sigma) was added and incubated for 15 min, after three times washing, and 1,4-Diazabicycle [2.2.2] octance (Sigma) was added in LTCs. The LTCs were observed using confocal laser scanning microscope (LSM880, Carl Zeiss, Jena, Germany).

### 2.10. Cell to Cell Adhesion Assay

The two cultivated dishes containing LTCs were prepared for cell to cell adhesion assay. After 24 h, one monolayer of LTC was washed with twice DMEM supplemented 0.1% BSA and another LTC was dissociated by 0.25% trypsin-EDTA which was incubated with DMEM supplemented 0.1% BSA and 1.0 μM Celltracker Red CMTPX (Thermo Fisher) for 30 min at 37 °C in the dark room. Then cell suspensions were washed twice with DMEM supplemented 0.1% BSA at 400× *g* for 5 min. Incubated LTCs with Celltracker Red were seeded on monolayer LTCs, then the cells were incubated with phenol free D/F12 supplemented 0.1% BSA, 1% P/S and 0.01 mM PGF2α. After 24 h, the LTCs were gently washed twice with DMEM supplemented 1% BSA and observed by fluorescence microscopy (BX-50, Olympus). The fluorescent LTC images were collected using digital camera system (EOS750D, Canon, Tokyo, Japan), the images were converted to 8 bit and controlled to detect red-stained LTCs by threshold filter. The red fluorescence stained cells were considered as attached LTCs on monolayer cells. The number of attached cells were counted using the analyze particles function of the ImageJ software.

### 2.11. Statistical Analysis

Data were analyzed using SAS ver. 9.4 software (SAS Institute, Cary, NC, USA). Data are presented as mean ± standard error mean. Intensity of the Rho/ROCK protein expression and number of attached LTCs were normalized to 0 mM PGF2α-induced group (control). The values of 0 mM PGF2α-induced group (*n* = 5) and 0.01 mM PGF2α-induced group (*n* = 5) were compared using Student’s t-test. A *p*-value < 0.05 was considered statistically significant.

## 3. Results

### 3.1. Location of LGCs and LTCs in CL Tissue

**Figure 2:**
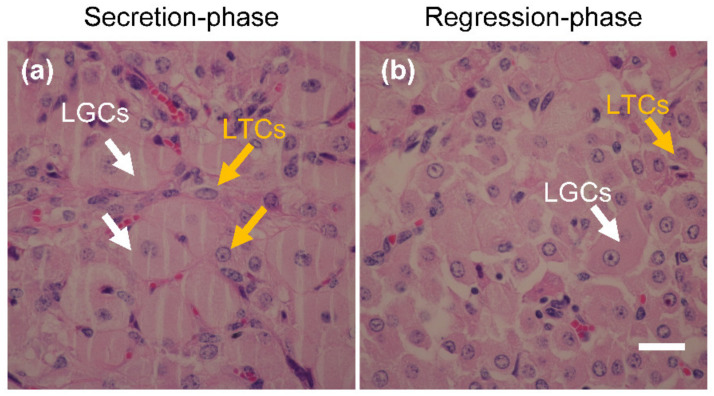
Histological images of bovine corpus luteum (CL) section. The CL sections were stained using Eosin-Y and hematoxylin method, secretion-(**a**) and regression (**b**)-phases CL were isolated from heifer. White bar: 25 μm.

The morphology of secretion and regression phase bovine CL sections is shown in Figure 2. Normal morphology of LGCs (Figure 2a, white arrows) and LTCs (Figure 2a, yellow arrows) were observed in secretion-phase bovine CL. The diameters of the LTCs were smaller than those of LGCs, and the LTCs were between the LGCs and LCs. However, the morphology of the LGCs (Figure 2b, white arrows) and LTCs (Figure 2b, yellow arrows) were mostly disrupted in regression-phase CL, and the cell-cell distances between each LC were increased in regression-phase (Figure 2b) compared to those of secretion-phase (Figure 2a) in bovine CL.

### 3.2. Detection of the LTC Subpopulation by Flow Cytometry

The schema of LTC isolation and flow cytometric analysis are shown in Figure 1. Isolated LCs from secretion-phase CLs (Figure 1a) were attached in the culture dish (Figure 1b). The colony of LTCs (Figure 1f, white line area) was observed when seeding density of LCs was low (Figure 1f) and isolated LTCs, by cloning cylinder, were cultured in the form of a monolayer in the new culture dish (Figure 1g). The β-actin-positive LCs (Figure 1c,d red dots) were divided into three subpopulations: red blood cells (Figure 1d, yellow area), other luteal cells (Figure 1d, green area; LTCs, LECs, and other cells) and LGCs (Figure 1d, blue area). In contrast, β-actin-positive LTCs (Figure 1h,i, red dots) were detected in only one subpopulation (Figure 1i, black area). However, red blood cells also contain β-actin [43], and the ratio of the two, excluding for the red blood cell subpopulation, were 89.4 ± 1.2% (LTCs, LECs and other cells) and 10.6 ± 1.2% (LGCs) in the LC subpopulation (Figure 1e). In addition, the maximum peaks of the *x*-axis in one of the other luteal cells (Figure 1e, red arrow) and LTCs (Figure 1j, red arrow) were exactly matched in the FSC histogram.

### 3.3. TC Lineage, Steroidogenic and IF Marker Assays

**Figure 3:**
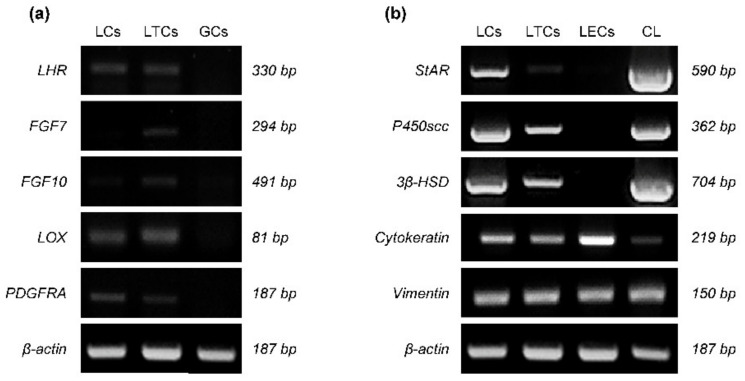
Expression of mRNA related with theca cells lineage markers (**a**), steroidogenic function and intermediate filament (**b**) in bovine luteal theca cells (LTCs). LCa: uteal cells, GCs: granulosa cells, LECs: luteal endothelial cells, CL: secretion-phase corpus luteum (CL), LHR: luteinizing hormone receptor, FGF7: fibroblast growth factor 7, LOX: lysyl oxidase, PDGFRA: platelet derived growth factor receptor alpha, StAR: steroidogenic acute regulatory protein, P450scc: cholesterol side-chain cleavage enzyme, and 3β-HSD: 3β-hydroxysteroid dehydrogenase.

The expression of luteinizing hormone receptor (LHR), fibroblast growth factor 7 (FGF7), FGF10, lysyl oxidase (LOX), and platelet-derived growth factor receptor alpha (PDGFRA) mRNA is shown in Figure 3a. The expression of LHR, FGF7, FGF10, LOX and PDGFRA mRNA were detected in LCs and LTCs, but expression of these mRNAs was not observed in GCs (Figure 3a). Steroidogenic acute regulatory protein (StAR), cholesterol side-chain cleavage enzyme (P450scc), and 3β-hydroxysteroid dehydrogenase (3β-HSD) mRNAs were expressed in LCs and LTCs, but not in LECs (Figure 3b). In addition, expression of *StAR*, *P450scc* and *3β-HSD* mRNA were less in LTCs compared to LCs (Figure 3b). Cytokeratin and vimentin mRNAs were expressed in LCs, LTCs and LECs (Figure 3b).

### 3.4. The Distribution of Cytokeratin and Vimentin Proteins in PGF2α-Induced Bovine LTCs

**Figure 4:**
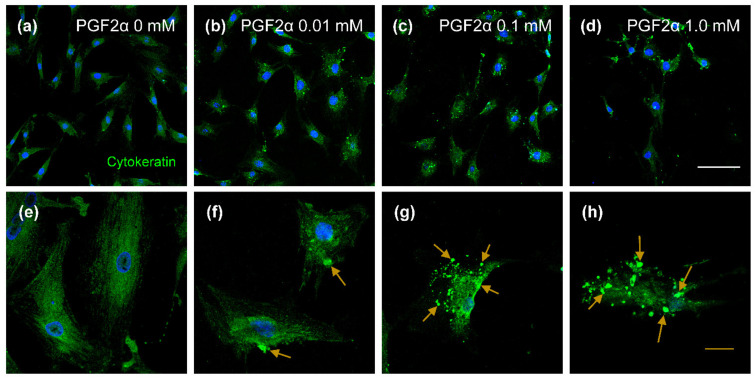
Confocal laser scanning microscope image of cytokeratin protein in prostaglandin F2 alpha (PGF2α)-induced bovine luteal thecal cells (LTCs). The LTCs were incubated in culture media supplemented 0 mM (**a**,**e**), 0.01 mM (**b**,**f**), 0.1 mM (**c**,**g**), and 1.0 mM (**d**,**h**) PGF2α. Green: cytokeratin protein, blue: nucleus, yellow arrows: damaged cytokeratin, white line: 50 μm, yellow line: 20 μm.

**Figure 5:**
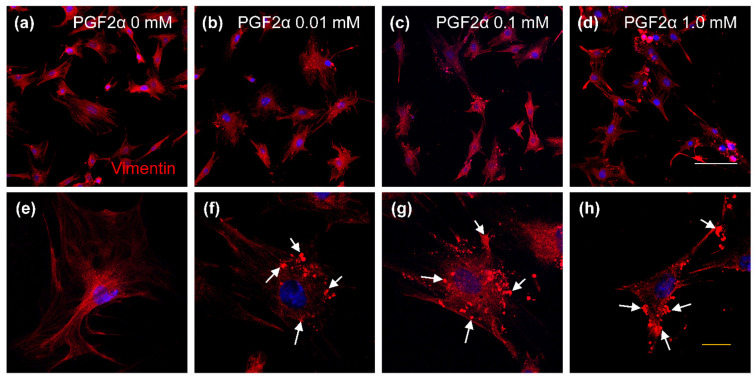
Confocal laser scanning microscope image of vimentin protein in prostaglandin F2 alpha (PGF2α)-induced bovine luteal thecal cells (LTCs). The LTCs were incubated in culture media supplemented 0 mM (**a**,**e**), 0.01 mM (**b**,**f**), 0.1 mM (**c**,**g**), and 1.0 mM (**d**,**h**) PGF2α. Green: vimentin protein, blue: nucleus, white arrows: damaged vimentin, white line: 50 μm, yellow line: 20 μm.

The expressio of cytokeratin protein is shown in Figure 4. The cytokeratin proteins were disrupted in the 0.01 mM (Figure 4b,f), 0.1 mM (Figure 4c,g) and 1.0 mM (Figure 4d,h) PGF2α-inducedc LTC groups compared with those of the control group (0 mM PGF2α; Figure 4a,e). In addition, aggregated cytokeratin proteins were observed in PGF2α-induced LTCs (Figure 4f–h, yellow arrows). The vimentin proteins were normally expressed in bovine LTCs (Figure 5); similar to cytokeratin, vimentin proteins in LTCs were also damaged by PGF2α (Figure 5b–d,f–h, white arrows), but aggregated vimentin protins were not detected in the control group (Figure 5a,e). Cytokeratin and vimentin proteins were normally distruibuted in the cytoplasm of bovine LTCs (Figure 4a and Figure 5a).

### 3.5. Effect of PGF2α on Rho/ROCK and Desmoplakin Expression in Bovine LTCs

**Figure 6:**
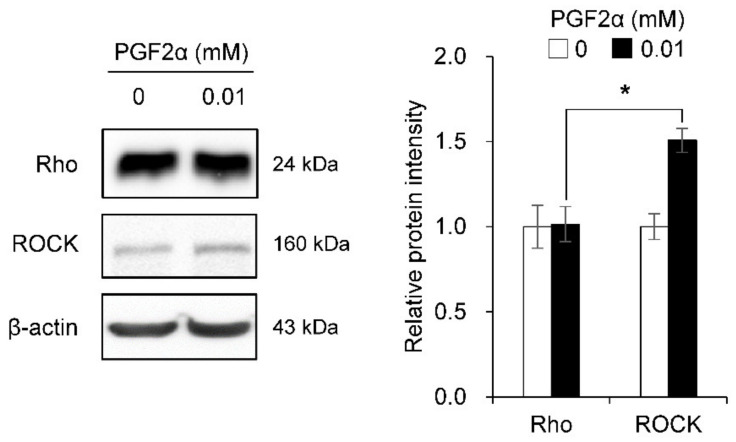
Influence of prostaglandin F2 alpha (PGF2α) on Rho and Rho-associated protein kinase (ROCK) protein in bovine luteal theca cells (LTCs). The protein was analyzed using western blotting. * *p* < 0.05, *n* = 5.

**Figure 7:**
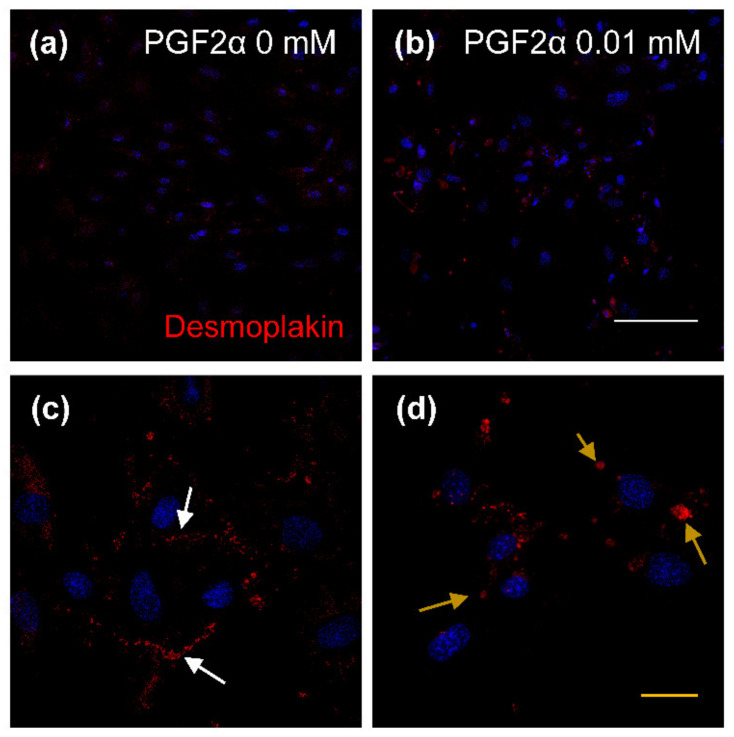
Expression of desmoplakin in prostaglandin F2 alpha (PGF2α)-induced bovine luteal thecal cells (LTCs). The LTCs were observed using confocal laser scanning microscope. The LTCs were incubated with 0 mM (**a**,**c**) and 0.01 mM (**b**,**d**) PGF2α for 24 h, white arrows: normal desmoplakin, yellow arrows: damaged desmoplakin, white line: 50 μm, yellow line: 20 μm.

The influence of PGF2α on Rho and ROCK protein expression is shown in Figure 6. Rho protein was not significantly different between 0 and 0.01 mM PGF2α-induced LTCs, whereas ROCK protein was increased (*p* < 0.05) in bovine LTCs in the 0.01 mM PGF2α treatment group compared to that of the control group (0 mM PGF2α). The distribution of desmoplakin in bovine LTCs is shown in Figure 7. The desmoplakin proteins were normally arranged around the plasma membrane in LTCs (Figure 7c, white arrows). However, protein of desmoplakin was aggregated and irregularly observed in the 0.01 mM PGF2α-induced bovine LTCs (Figure 7d, yellow arrows).

### 3.6. Influence of PGF2α on the Cell–Cell Adhesion Ability in Bovine LTCs

**Figure 8:**
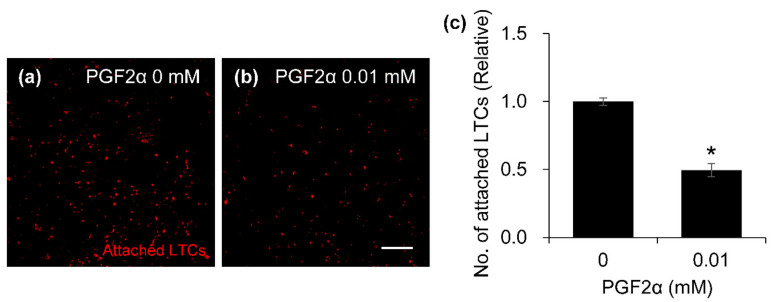
Effect of prostaglandin F2 alpha (PGF2α) on cell–cell adhesion ability in bovine luteal theca cells (LTCs). The cell-tracker Red stained LTCs were incubated with 0 mM (**a**) and 0.01 mM (**b**) PGF2α on the monolayer LTCs. The attached LTCs ((**a**,**b**), red dots) on the monolayer cells were counted using the segmentation tools of ImageJ, and the number of attached cells was normalized to 0 M PGF2α treatment (**c**). * *p* < 0.05, *n* = 5.

The cell–cell adhesion ability of the PGF2α-induced bovine LTCs is shown in Figure 8. The 0 and 0.01 mM PGF2α-induced LTCs were attached to a monolayer of LTCs (Figure 8a,b, red). In addition, the number of attached LTCs on monolayer cells was significantly decreased in 0.01 mM PGF2α-induced LTCs compared to that of untreated PGF2α LTCs (*p* < 0.05).

## 4. Discussion

It is well known that there are morphologically diverse LSCs and LECs in bovine CL, and the proportion the number of cells is larger for LTCs than LGCs, but the volume of LGCs is approximately 13 times larger than that of LTCs [2]. Based on these morphological characteristics of LCs, it was confirmed that large and small LSC cell populations were clearly divided according to dimeters in LCs isolated from sheep and cows [9,10]. Similarly, the results of flow cytometry in the current study showed that β-actin-positive LCs isolated from secretion-phase CLs were largely divided into three subpopulations. In addition, there were two FSC peaks for other luteal cells (LTCs, LECs and other cells), which suggests that other luteal cell subpopulations contain more than two types of cell. We also successfully isolated LTCs from LCs according to their morphology characteristics and confirmed that the maximum values of *X*-axis FSC peaks in LTCs and other cell subpopulations containing LTCs matched exactly. In addition, to determine the origins of GCs and TCs, LTC membrane surface markers [44] and TC-specific molecular markers such as *FGF7* and *FGF10* [45] were analyzed, and there was stronger mRNA expression in TCs and LTCs than in GCs and LGCs of genes such as *LOX* and *PDGFRA* [46]. Furthermore, the expression of steroidogenic factors (*StAR*, *P450scc* and *3β-HSD*) in LTCs was lower than that in LGCs. The results of marker assay in this study shows that the LTCs originated from TCs and participate in less P4 production than LGCs, and the expression of cytokeratin and vimentin in bovine LTCs indicates that the IFs are present in LTCs. In general, the normal expression of vimentin in TCs from mesenchymal stem cells has been well known through other studies [16,47], whereas the study of cytokeratin in bovine LTCs has not been clearly demonstrated. Moreover, studies on cytokeratin protein in bovine LCs reported that cytokeratin 8/18 protein was normally expressed in LCs collected from bovine proliferation- and regression-phase CL tissues, and previous studies used a heterogeneous luteal cell mixture derived from bovine CL tissues [18,48]. Our study confirmed cytokeratin expression in bovine LTCs.

Direct degradation of LSCs is caused by cytokines, nitric oxide (NO), leukotriene C4, and endothelin-1, and mechanisms of bovine LSC degradation by direct PGF2α administration have not been clearly elucidated [33,49]. The reason for the lack of studies on LSC regression by direct PGF2α administration is that PGF2α is known to indirectly activate apoptosis of LSCs via activating tumor necrosis factor-α (TNFα), interferon-γ (IFNγ), NO and endothelin-1 of lymphocytes, macrophages and LECs [50]. In general, studies on the influence of PGF2α in bovine LSCs reported that 0.01 and 0.001 mM PGF2α increased P4 production in bovine LSCs, and 0.01 mM PGF2α is known to increase intracellular Ca^2+^ (cellular secondary messenger) [41,42]. In summary, the previous studies of PGF2α-mediated effects on bovine LSCs mainly used PGF2α to induce LSC degeneration with minimum stimulation [41,42,51,52,53,54]. Interestingly, treatment of bovine LSCs with 1.0 μg/mL synthetic PGF2α (2.8 μM dinoprost, 2.4 μM cloprostenol and 2.2 μM luprostiol) for 24 h resulted in decreased cell viability, increased DNA fragmentation, and caspase-3 activity [42], and a previous study means that a single PGF2α treatment directly affects bovine LTC degeneration. Therefore, based on previous studies regarding single PGF2α treatment of bovine LSCs, 0.01 mM (minimum concentration) was used for the present study to disrupt cell–cell adhesion in bovine LTCs.

To date, there have been no studies on the effects of PGF2α on IFs and desmosomes in bovine LSCs. Some studies on IFs in bovine LCs reported that cytokeratin 8/18 was decreased in the LCs derived from the regression-phase CL; additionally, Fas protein was increased in cytokeratin-positive LCs damaged by acrylamide [18,48]. Although the level of PGF2α is increased in regression-phase CL, this is still not enough to verify the relationship between PGF2α and IFs. Furthermore, changes in IF protein in LTCs were primarily demonstrated in this study, and LTCs also had depolymerized cytokeratin and vimentin IFs at concentrations over 0.01 mM PGF2α. The results showed that direct PGF2α can disrupt IFs in LTCs, even though it is not one of the cytokines, NO, leukotriene C4 and endothelin-1 that are known to cause apoptosis of LCs [33,49]. Thus, at concentrations over 0.01 mM PGF2α could directly regress bovine LTCs via disruption of cytokeratin and vimentin IFs, possibly controlling cellular signaling involved in the cytoskeleton.

Rho, one of the Ras superfamily members, activates ROCK, controls cytoskeletal organization, such as actin filaments and IFs, and it has been reported that PGF2α activates Rho protein signaling through PGF2αR, one of the GPCRs in HEK-293 cells; however, studies on bovine LCs have not yet been done. Our study focused on the Rho/ROCK signals that regulate IFs such as cytokeratin and vimentin in LTCs and confirmed that the IFs were disrupted when ROCK protein was increased. Thus, we determined that single administration of PGF2α (over 0.01 mM) directly increased ROCK and affected the disruption of cytokeratin and vimentin in LTCs, and we hypothesized that PGF2α may disconnect cell–cell desmosome adhesions.

The desmoplakin is an anchoring plaque connecting CAMs (desmocollin and desmoglein) and IFs and is an essential organelle for desmosomal adhesion [55]. Studies on desmoplakin-null cells have reported that desmoplakin plays a significant role in the connection between cytokeratin, vimentin, and cell adhesion in mammals [56,57]. Additionally, studies on the structure and adhesion of tumor cells through suppression of the desmosome system have been actively carried out in the oncology field [58]. Likewise, our study showed that cytokeratin and vimentin IFs were disrupted and that the desmoplakin arrangement was also disturbed by PGF2α in bovine LTCs. In addition, it was confirmed that LTC–LTC adhesion ability was also reduced by PGF2α; PGF2α had a negative influence on the cytoskeleton and cell–cell adhesion in bovine LTCs. Unfortunately, in the present study, we did not investigate the association between apoptotic signals and IF damage caused by PGF2α; however, we will investigate the relationship between Rho/ROCK, IFs, and cell-cell adhesion by PGF2α without apoptotic signals in bovine LTCs.

From a histological perspective, because LTCs exist between LGCs, LECs and other cells, we hypothesized that LTCs may be involved in the adhesion of heterogeneous cells in CL tissues and investigated the distribution of cytokeratin and vimentin IFs and desmoplakin protein by PGF2α- and Rho/ROCK-mediated changes in GPCR activation. Consequently, PGF2α not only activated ROCK but also disrupted IF proteins (cytokeratin and vimentin), desmoplakin and cell-cell adhesion. For several decades, even though apoptosis by cytokines, NO, leukotriene C4 and endothelin-1 have been the focus of structural regression of bovine CL.

Based on our results, we would like to suggest a study on G-protein activation via PGF2α-GPCR stimulation and disruption of cytoskeleton-cell adhesion in bovine CL. It is well known that the extrinsic and intrinsic apoptotic signaling cascades adversely affect small G-proteins and cause cytoskeleton disruption. Nevertheless, if the direct influence of PGF2α-induced small G-protein, cytoskeleton and cell-cell adhesion is more thoroughly studied in bovine LTCs, the results could be helpful in the study of cancer and tumor therapy regarding the disruption of the cytoskeleton and cell adhesion in ovarian tumors using selective cells.

## Figures and Tables

**Table 1:** Primer information.

Gene	Primer (5′–3′)	Product Size (bp)	Annealing Temp. (°C)	Cycles	Accession No.
*LHR*	F: CTCAAGCTTTCAGAGGACTT	224	60	30	U87230
	R: TCTGGAAGCTTGTGGATGCCTG				
*FGF7*	F: CTGCCAAGTTTGCTCTACAG	294	55	24	S72475
	R: TCCAACTGCCAGGGTCCTGAT				
*FGF10*	F: CTTCTTGGTGTCTTCCGTCC	491	65	30	AF213396
	R: CTCCTTTTCCATTCAATGCC				
*LOX*	F: CGTCCGCTGTGAAATTCGCT	81	60	21	NM_173932.4
	R: TGGCTTGCTTTCTAATACGGTGA				
*PDGFRA*	F: GAGTGAAGTGAGCTGGCAGT	187	60	21	XM_010806122.2
	R: CTGCCCTCGATCTCGTTCTC				
*StAR*	F: CATGGTGCTCCGCCCCTTGGCT	590	60	24	BC110213
	R: CATTGCCCACAGACCTCTTGA				
*P450scc*	F: AACGTCCCTCCAGAACTGTACC	362	60	24	BC133389
	R: CTTGCTTATGTCTCCCTCTGCC				
*3β-HSD*	F: TCCACACCAGCACCATAGAA	704	60	21	BC111203
	R: CTCCTTGGTTTTCTGCTTGG				
*Cytokeratin*	F: GAGGAGCTGAACAGGGAGGT	219	60	24	NM_001015600
	R: CTGGGCTTCGATACCACTGA				
*Vimentin*	F: AAGCCGAGAGCACTCTGCAGTCT	150	60	21	NM_173969.3
	R: GGGCCTGAAGCTCCTGGATTTCCT				
*PGF2αR*	F: TTAGAAGTCAGCAGCACAG	519	60	30	D17395
	R: ACTATCTGGGTGAGGGCTGATT				
*β-actin*	F: GAAGATCTGGCACCACAC	187	60	24	AY141970
	R: AGAGGCATACAGGGACAGC				

## Data Availability

Data are contained within the article and Supplementary Material. Raw data are available on request from the corresponding author.

## Supplementary Materials

The following are available online at https://www.mdpi.com/article/10.3390/ani11041073/s1.
