# Supplementary material for: Prostaglandin F2 Alpha Triggers the Disruption of Cell Adhesion with Cytokeratin and Vimentin in Bovine Luteal Theca Cells
Source: Animals (Basel). 2021 Apr 9;11(4):1073. doi: 10.3390/ani11041073 (PMC8069824; doi:10.3390/ani11041073)
Supplement: Supplementary file 1 [file animals-11-01073-s001.pdf]

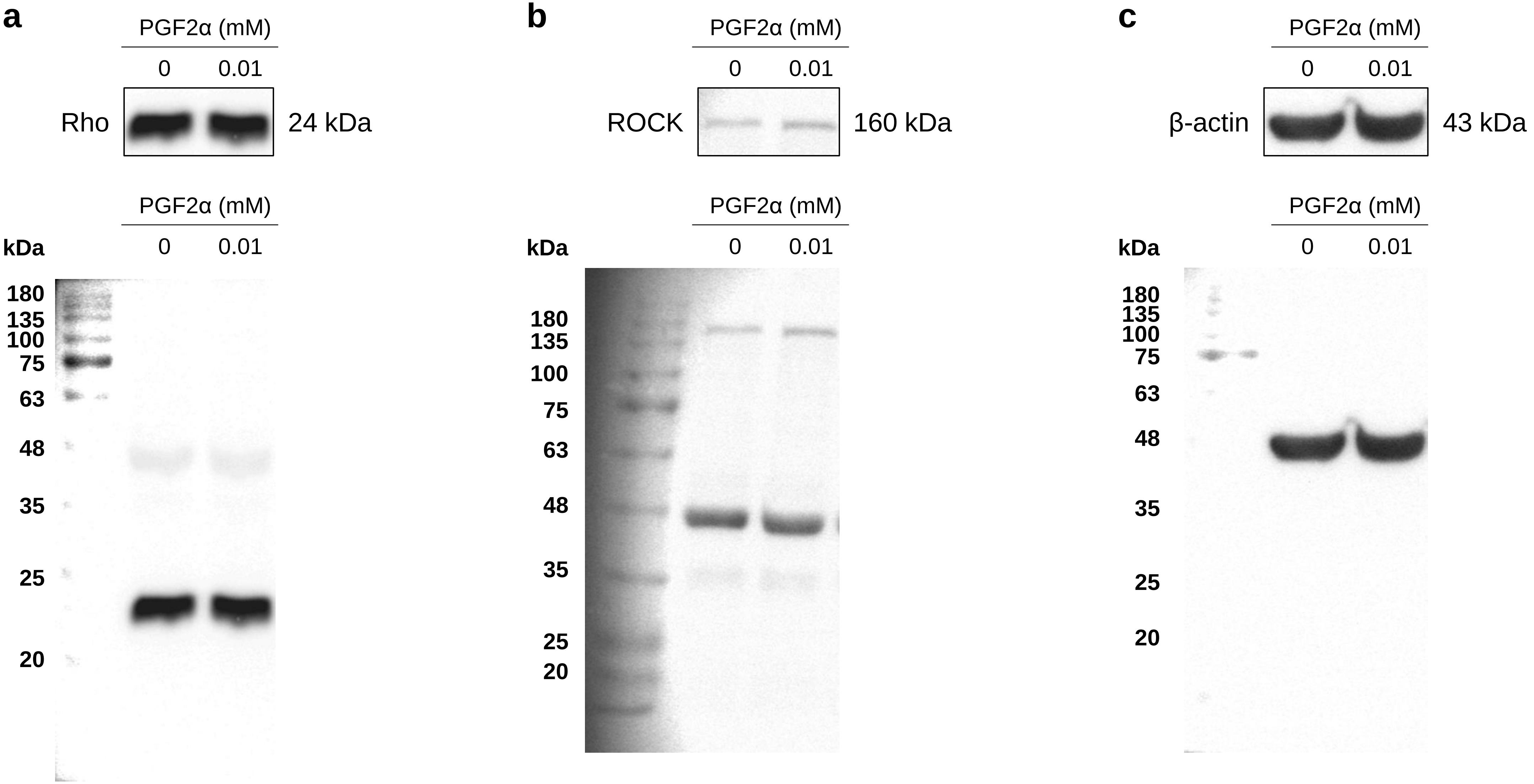

Supplementary figure 1. Influence of prostaglandin F2 alpha (PGF2α) on Rho (a) and Rho-associated protein kinase (ROCK, b) protein in bovine luteal theca cells (LTCs). Rho and ROCK proteins were normalized by β-actin (c). This data are corresponded in Figure 6 of manuscript.
